# Linking metacercariae and adults of *Microphallus basodactylophallus* (Digenea: Microphallidae), based on larval stages from ctenophores and adult parasites from aquatic birds found in Mexico

**DOI:** 10.1007/s11230-023-10131-2

**Published:** 2023-12-21

**Authors:** Yeraldin Aldama-Prieto, Jorge L. Navarro-Serralde, Enrico Alejandro Ruíz, Ana L. Sereno-Uribe, Martín García-Varela

**Affiliations:** 1 Departamento de Zoología, Instituto de Biología, Universidad Nacional Autónoma de México, Ciudad Universitaria, C. P. 04510 Mexico, Mexico; 2 Laboratorio de Ecología, Departamento de Zoología, Escuela Nacional de Ciencias Biológicas, Instituto Politécnico Nacional, Mexico, C. P. 11340 Mexico

## Abstract

Members of the genus *Microphallus* Ward, 1901, are endoparasites mainly of birds and mammals distributed worldwide. Unencysted metacercariae of *Microphallus* sp., were collected from the mesoglea of ctenophores of the genus *Pleurobrachia* Fleming; adult digeneans were recovered from the intestines of *Eudocimus albus* Linnaeus (Threskiornithidae) and *Buteogallus urubitinga* Gmelin (Accipitridae), in four locations from southeastern Mexico. Adult specimens were identified as *M. basodactylophallus* (Bridgman, 1969) based on the following features: body pyriform entirely covered by minute spines, prepharynx short, oesophagus very long, caeca short and widely divergent, testes slightly symmetrical and excretory vesicle short and V-shaped. Sequences from D1–D3 domain of the large subunit of ribosomal DNA (LSU) were generated, aligned, and compared with those of congeneric species available in GenBank. Phylogenetic analyses indicated that the metacercariae and adults formed a clade together with an isolate identified as *M. basodactylophallus* from Florida, USA (GenBank: AY220628). The intraspecific genetic divergence among isolates was low ranged from 0.0% to 0.6%, allowing the link between the two stages of the life cycle. We observed phenotypic plasticity in the morphological traits of *M. basodactylophallus* adults in definitive hosts (mammals and birds) throughout the distribution, which ranged from the USA to southeastern Mexico. Finally, the unencysted metacercariae identified as *M. basodactylophallus* represent the first report of a microphallid in ctenophores.

## Introduction

Digeneans are the most diverse group of parasites recorded in wildlife from Mexico. To date, 624 species have been recorded and classified into 311 genera and 78 families with a high percentage of endemicity, representing 30% of the biodiversity of digeneas. However, the inventory of digeneans in the country is far from complete (Pérez-Ponce de León et al. 2007), and Mexico could be considered a hotspot of diversity because it occupies a transitional position between Nearctic and Neotropical biogeographical regions (Morrone, 2006; Pérez-Ponce de León et al., 2007). The genus *Microphallus* Ward, 1901 contains approximately 45 species distributed worldwide parasitizing birds and occasionally mammals (Deblock, 2008; Galaktionov & Blasco-Costa, 2018). In Mexico, metacercariae of *Microphallus* sp. were recorded in the bulldog goodeid (*Alloophorus robustus* Bean) from lakes of central Mexico. However, the records could not be verified because the specimens are not available in any collection (see Pérez-Ponce de León et al., 2007). Two species of *Microphallus* were described from Mexico, i.e., *M. muellhaupti* Coil, 1955, from a shorebird (*Tringa* sp.) and *M. trilobatus* Cable and Kuntz, 1951, from a roadside hawk (*Rupornis magnirostris* Gmelin) (see Pérez-Ponce de León et al., 2007).

The species *M. basodactylophallus* (Bridgman, 1969) Deblock, 1971, was described from the intestine of a racoon (*Procyon lotor* Linnaeus) in North America (see Bridgman, 1969), and since then, this microphallid has been recorded in other definitive hosts, the marsh rice rat (*Oryzomys palustris* Harlan) (Heard & Overstreet, 1983). The life cycle of *M. basodactylophallus* is well known; adult worms live and reproduce sexually in the digestive tract of their definitive hosts. Mature eggs are expelled in the environment with the faeces of the definitive host. Later, a gastropod mollusc serves as the first intermediate host, and crabs, crayfishes, and shrimp serve as second intermediate hosts, which are eaten by the definitive hosts, completing the life cycle (see Bridgman, 1969; Heard & Overstreet, 1983; Gehman et al., 2021).

During a study of parasites infecting invertebrates and vertebrates from southeastern Mexico, unencysted metacercariae of *Microphallus* sp., were found in the mesoglea of ctenophores (*Pleurobrachia* sp.) at a single locality, and adult specimens were recovered from the intestine of two bird species at three localities. A detailed morphological study of both stages and comparative analyses based on sequences of domains D1–D3 of the large subunit of ribosomal DNA allowed the link between these two stages. The current study provides a morphological and molecular characterization of the larval and adult specimens identified as *M. basodactylophallus,* a generalist parasite of mammals and birds distributed from the USA to southeastern Mexico.

## Materials and methods

**Fig. 1:**
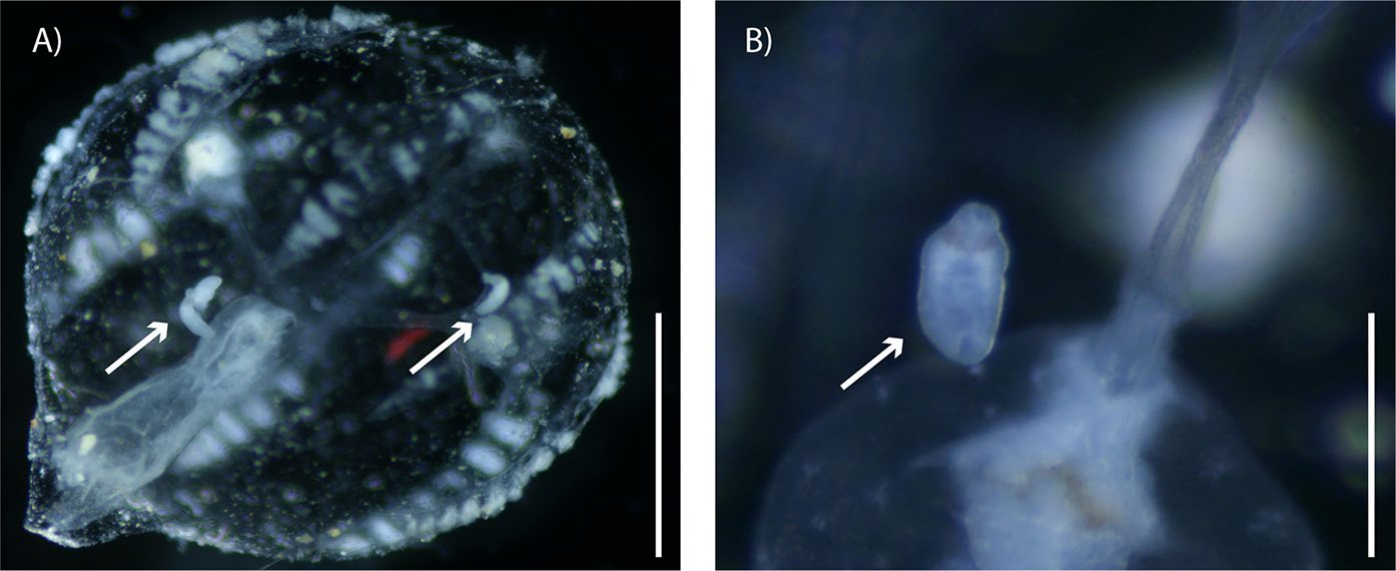
Photographs of a ctenophore (*Pleurobrachia* sp.) with unencysted metacercariae of *Microphallus basodactylophallus* (**A**); Unencysted metacercaria in the mesoglea (**B**). Arrow indicates the metacercariae*;* Scale bars = 1 mm (**A**); 0.5 mm (**B**)

A total of 376 ctenophores (*Pleurobrachia* sp.) were collected in Tampamachoco, Veracruz (20° 58′ 25.4″ N; 97° 20′ 22.2″ W). Nine birds of two species were collected at three localities in southeastern Mexico: white ibises (n = 2) (*Eudocimus albus* Linnaeus) in Tlacotalpan, Veracruz (18° 37′ 04.15″ N; 95° 38′ 56.10″ W), and great black hawks (n = 3) (*Buteogallus urubitinga* Gmelin) in Playa Paraiso, Tabasco (18° 24′ 25.31″ N; 93° 29′ 50.38″ W), and Tupilco, Tabasco (n = 4) (18° 26′ 6.004″ N; 93° 7′ 44.60″ W). Each ctenophore was placed in a plastic tube containing marine water and 2% formol. Then, each ctenophore was identified following the keys of Mills & Haddock (2007). Birds were identified following Howell & Webb (1995) and the American Ornithologists’ Union (1998) guidelines. Unencysted metacercariae were removed from the mesoglea of infected ctenophores, washed in water and placed in 100% ethanol (Fig. 1). Adult digeneans were removed from the intestine of the birds using a stereomicroscope (EZ4 Leica). Later, the collected parasites were relaxed in hot distilled water and preserved in 100% ethanol for morphological and molecular analyses.

### Morphological analyses

The specimens were stained with Mayer’s paracarmine (Merck, Darmstadt, Germany), dehydrated in an ethanol series, cleared in methyl salicylate and mounted in Canada balsam for morphological analysis. Specimens were then examined using a compound microscope equipped with a bright field (Leica DM 1000 LED microscope, Leica, Wetzlar, Germany). Measurements were taken using Leica Application Suite microscope software (Leica Microsystems GmbH, Wetzlar, Germany) and are given in micrometres as range followed by mean in parentheses. Some specimens from each locality were dehydrated in ethanol series, critical-point dried, sputter coated with gold, and examined with a Hitachi Stereoscan Model S-2469N scanning electron microscope operating at 15 kV. Adults and metacercariae were deposited in the Colección Nacional de Helmintos (CNHE) of the Instituto de Biología, Universidad Nacional Autónoma de México (UNAM), México City (CNHE. No. 12063–12066).

### Amplification and sequencing of DNA

**Fig. 2:**
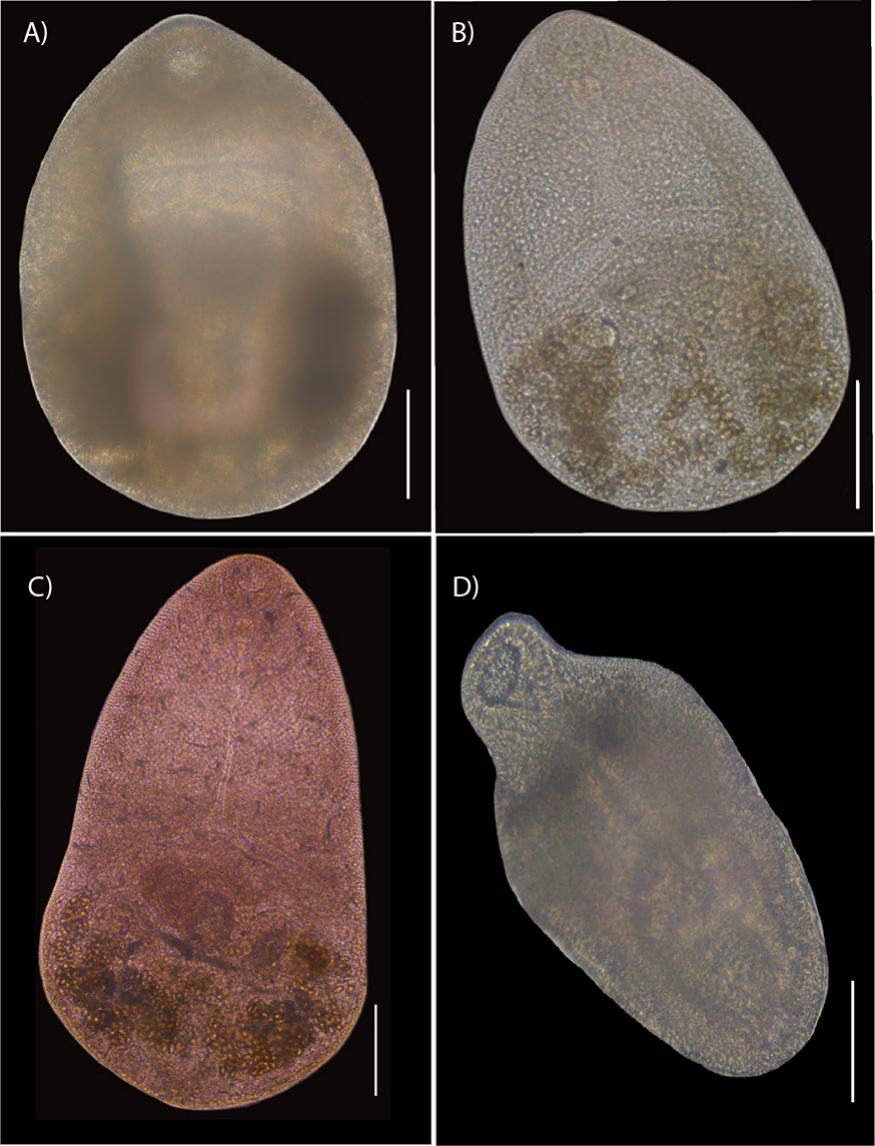
Photogenophores of *Microphallus basodactylophallus*. Adult forms (**A–C**); specimens collected from the intestine of *Buteogallus urubitinga* from Playa Paraiso, Tabasco (**A**) and from Tupilco, Tabasco (**B**); specimen collected from the intestine of *Eudocimus albus* from Tlacotalpan, Veracruz (**C**); unencysted metacercariae collected from the mesoglea of *Pleurobrachia* sp., from Tampamachoco, Veracruz (**D**); Scale bar = 100 μm

Prior to extraction of the genomic DNA, each specimen was mounted on a microscope slide, and images were taken as references with a bright field Leica DM 1000 LED microscope (Leica, Wetzlar, Germany). Each image was linked with its genomic DNA, known as a *photogenophore* (see Andrade-Gómez & García-Varela, 2021) (Fig. 2). Specimen were removed from the microscope slide, placed alone in a tube and digested overnight at 56 °C. The digestion solution contained 10 mM Tris-HCl (pH 7.6), 20 mM NaCl, 100 mM Na_2_ EDTA (pH 8.0), 1% sarcosyl, and 0.1 mg/ml proteinase K. Following digestion, DNA was extracted from the supernatant using DNAzol reagent (Molecular Research Center, Cincinnati, Ohio) according to the manufacturer’s instructions. The D1–D3 domains of the LSU of rDNA were amplified using the forward 391, 5′-AGCGGAGGAAAAGAAACTAA-3′ (Nadler et al., 2000), and the reverse 536, 5′-CAGCTATCCTGAGGGAAAC-3′ (García-Varela & Nadler, 2005). PCRs (25 µl) consisted of 1 µl of each primer (10 µM), 2.5 µl of 10x PCR Rxn buffer, 1.5 µl of 2 mM MgCl_2_, 0.5 µl of dNTPs (10 mM), 16.375 µl of water, 2 µl of genomic DNA and 1 U of Taq DNA polymerase (Platinum Taq, Invitrogen Corporation, São Paulo, Brazil). The PCR cycling parameters for rDNA amplification included denaturation at 94 °C for 1 min, followed by 35 cycles at 94 °C for 1 min, annealing at 50 °C for 1 min, and extension at 72 °C for 1 min, with postamplification incubation at 72 °C for 10 min. Sequencing reactions were performed using the initial primers plus two internal primers, 502 (5′-CAAGTACCGTGAGGGAAAGTTGC-3′) and 503 (5′-CCTTGGTCCGTGTTTCAAGACG-3′) (García-Varela & Nadler 2005), with ABI Big Dye (Applied Biosystems, Boston, Massachusetts) terminator sequencing chemistry, and reaction products were separated and detected using an ABI 3730 capillary DNA sequencer. Contigs were assembled and base-calling differences were resolved using Codoncode Aligner version 9.0.1 (Codoncode Corporation, Dedham, Massachusetts).

### Alignments and phylogenetic analyses

LSU sequences obtained in the current research were aligned separately with sequences from other microphallid species downloaded from the GenBank database, and the species *Haematoloechus longiplexus* (Staffford, 1902) (AF387801), *Plagiorchis vespertilionis* (Müller, 1780) Braun, 1900 (AF151931) and *Telorchis assula* (Dujardin, 1845) Dollfus, 1957 (AF151915) were used as outgroups. The alignment was performed using the software Clustal W (Thompson et al., 1994) with default parameters and adjusted manually with the software Mesquite (Maddison & Maddison, 2011). The alignment consisted of 43 sequences with 1,279 nucleotides. The best model of nucleotide substitution was estimated with the Akaike information criterion (AIC) implemented in jModelTest v0.1.1 (Posada, 2008). The phylogenetic analyses were performed using maximum likelihood (ML) and Bayesian inference (BI) methods. ML was carried out with RAxML version 7.0.4 (Silvestro & Michalak, 2011), and Bayesian inference (BI) analyses were performed with MrBayes version 3.2.7 (Huelsenbeck & Ronquist 2001) using the Cyberinfrastructure with Phylogenetic Research (CIPRES) Science Gateway v3.3 online interface (Miller et al., 2010). To support each node, 10,000 bootstrap replicates were run with ML. BI analyses included Markov chain Monte Carlo (MCMC) searches with two simultaneous runs for 10 million generations, sampling every 1,000 generations, a heating parameter value of 0.2 and a “burn-in” of 25%. Trees were visualized in FigTree v.1.3.1 (Rambaut, 2010). The genetic divergence among taxa was estimated using uncorrected “p” distances with the program MEGA version 11 (Tamura et al., 2021).

## Results

### Morphological identification

Adult digeneas found in two bird species in southeastern Mexico were identified as *M. basodactylophallus,* based on similar morphology present in the original description of the species, by Bridgman (1969), which includes, (i) body pyriform; (ii) tegument covered entirely with minute spines; (iii) prepharynx short or absent; (iv) oesophagus very long; (v) caeca short, widely divergent; (vi) testes slightly symmetrical; and (vii) excretory vesicle short, V-shaped. The present specimens were somewhat divergent form the type material of

*M. basodactylophallus* regarding the morphometry of body, oral sucker, ventral sucker, pharynx, oesophagus, testes and eggs (see Table 1).

**Table 1:** Comparative metrical data of *Microphallus basodactylophallus*; number of host examined/infected (prevalence of infection)

Host	*Buteogallus urubitinga* n = 4 (2/4)	*Buteogallus urubitinga* n = 3 (1/3)	*Eudocimus albus* n = 2 (1/2)		*Rattus norvegicus albinus*
Locality	Tupilco, Tabasco.	Playa Paraíso, Tabasco	Tlacotalpan, Veracruz		Louisiana, USA
Source	Present study *n*=17	Present study *n* = 8	Present study *n* = 30	Total of specimens measured *n* = 55	(Bridgman, 1969) *n* = 10
Features	Range, Mean	Range, Mean	Range, Mean	Range, Mean	Range
Body length	344−407, 351	308−337, 327	460−640, 564	308−640, 414	415−455
Body maximum width	210−247, 219	171−197, 191	195−304, 237	171−304, 215	187−212
Oral sucker length (L)	49−58, 52	39−44, 41	45−71, 60	39−71, 51	50−62
Oral sucker width (W)	50−62, 52	42−49, 45	55−69, 63	42−69, 53	50−62
Ventral sucker L	48−68, 53	43−55, 50	46−70, 59	43−70, 54	52−65
Ventral sucker W	40−60, 44	37−50, 43	43−70, 54	37−70, 47	52−62
Prepharynx L	5−9, 7	2−6, 4	3−9, 8	2−9, 6	−
Pharynx L	22−35, 22	18−29, 25	21−32, 25	18−35, 24	22−25
Pharynx W	10−27, 22	12−24, 20	22−32, 26	10−32, 22	22−27
Oesophagus L	23−95, 72	30−78, 65	154−235, 187	23−235, 108	87−110
Right caecum L	94−190, 116	85−140, 124	96−163, 128	85−190, 122	125−152
Right caecum W	7−31, 16	12−25, 18	24−49, 38	7−24, 24	−
Left caecum L	97−134, 114	86−125, 111	90−110, 97	86−134, 107	−
Left caecum W	12−31, 22	15−28, 21	16−25, 21	12−31, 21	−
Mehlis gland L	53−64, 57	52−60, 57	54−69, 58	52−59, 57	−
Right Testis L	26−39, 34	29−35, 32	33−69, 50	26−69, 38	22−52
Right Testis W	21−50, 29	24−45, 34	43−67, 57	21−67, 40	65−95
Left Testis L	29−36, 33	31−33, 30	34−49, 44	29−49, 35	27−50
Left Testis W	23−48, 37	22−46, 39	46−57, 53	22−57, 43	50−95
Ovary L	40−64, 50	34−56, 50	40−73, 57	34−73, 52	52−75
Ovary W	35−76, 47	30−64, 57	37−73, 57	30−76, 53	50−75
Forebody L	192−312, 297	156−274, 232	293−396, 340	156−396, 289	187−212
Hindbody L	154−278, 254	145−263, 216	187−259, 222	145−278, 230	−
Egg L	9−20, 14	10−18, 13	9−18, 15	9−20, 14	16−17
Egg W	5−11, 8	5−12, 9	6−10, 8	5−12, 8	10−12

### Redescription

#### Adult

**Fig. 3:**
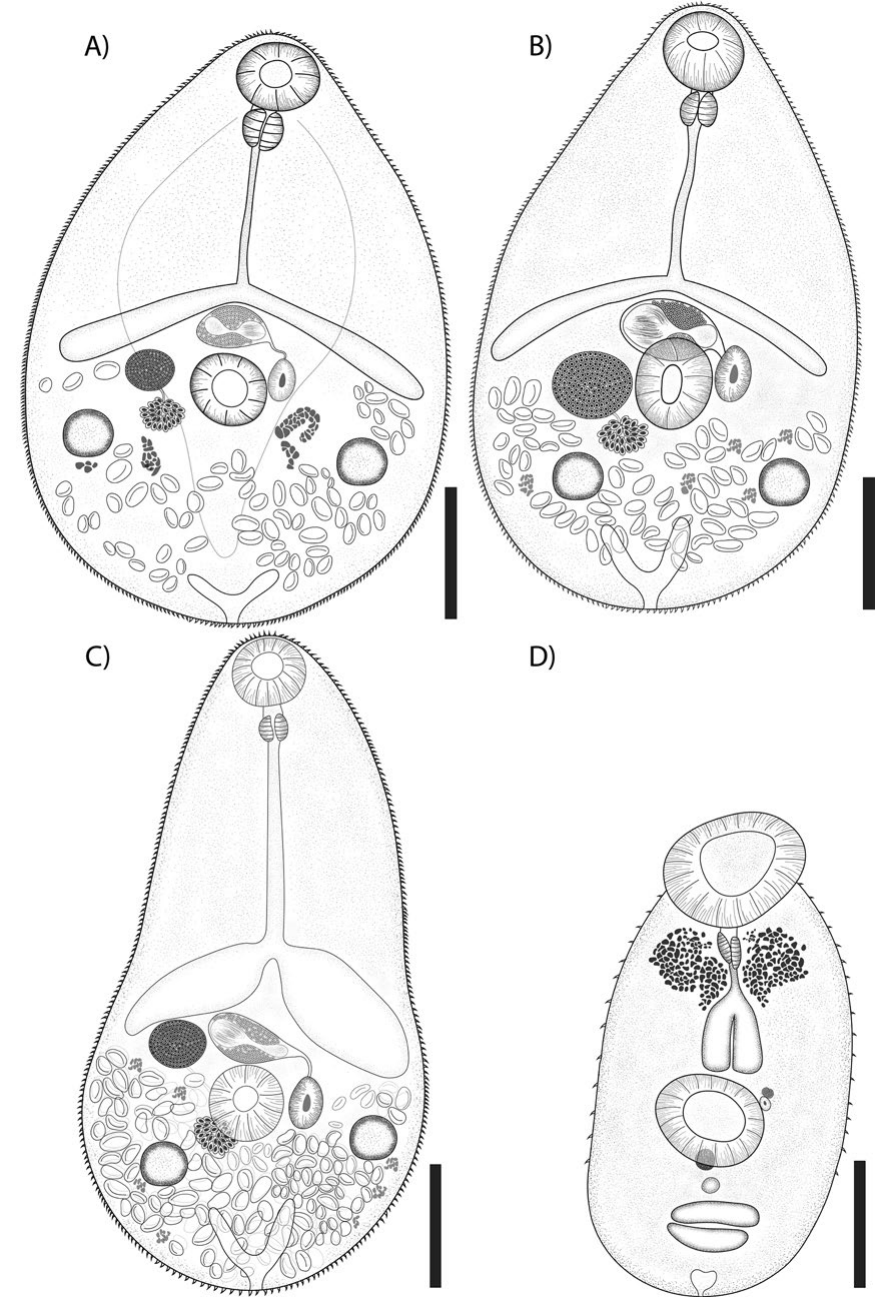
Drawings of *Microphallus basodactylophallus* whole worm voucher, ventral view*.* Adult forms (**A–C**); specimens collected from the intestine of *Buteogallus urubitinga* from Playa Paraiso, Tabasco (**A**) and from Tupilco, Tabasco (**B**); specimen collected from the intestine of *Eudocimus albus* from Tlacotalpan, Veracruz (**C**); unencysted metacercariae collected from the mesoglea of *Pleurobrachia* sp., from Tampamachoco, Veracruz (**D**); Scale bar = 100 μm

**Fig. 4:**
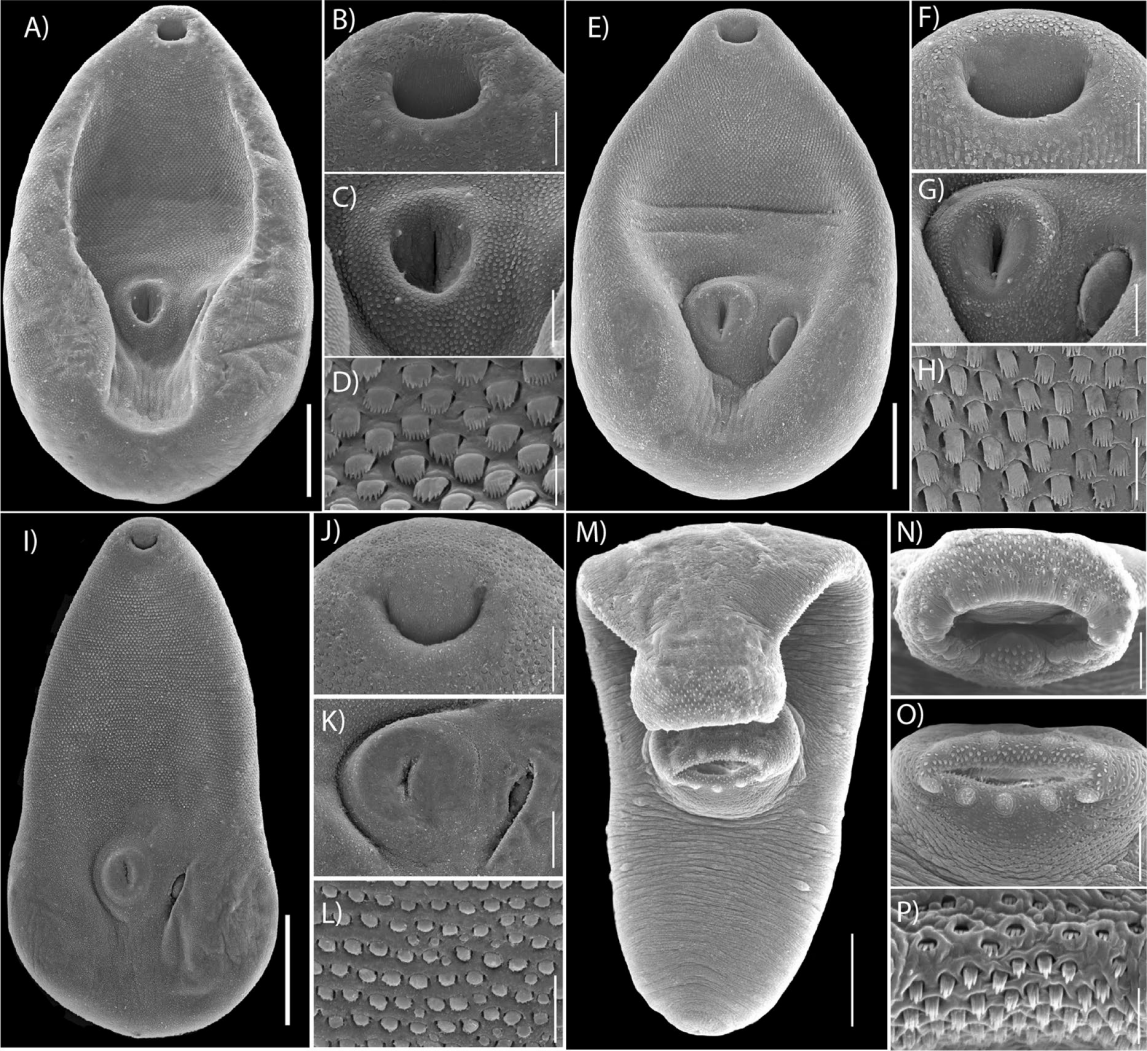
Scanning electron micrographs of *Microphallus basodactylophallus.* Adult forms (**A, E, I**); specimens collected from the intestine of *Buteogallus urubitinga* from Playa Paraiso, Tabasco (**A**) and from Tupilco, Tabasco (**E**); specimen collected from the intestine of *Eudocimus albus* from Tlacotalpan, Veracruz (**I**); unencysted metacercariae collected from the mesoglea of *Pleurobrachia* sp., from Tampamachoco, Veracruz (**M**); oral sucker (**B, F, J, N**); ventral sucker (**C, G, K, O**); tegumental spines (**D, H, L, P**). Scale bar = 100 μm (**A, E, I**); 30 μm (**B, C, F, G, J, K, N, O**); 5.00 μm (**D, H, L, P**); 50 μm (**M**).

[Measurements based on whole mounts of 55 specimens and 12 specimens for SEM (Figs. 3 and 4).]

Body dorsoventrally flattened, pyriform (Fig. 3A–C, Fig. 4A, E, I), with maximum width at level of ventral sucker 171–304. Body length 308–640. Tegument entirely covered with squamous minute spines with an average of 8 teeth on their distal margins (Fig. 4D, H, L). Forebody 156–396 long, representing 50–62% of body length. Oral sucker subterminal, subspherical, 39–71 × 42–69 (Fig. 3A–C; Fig. 4B, F, J). Ventral sucker equatorial or post-equatorial, subspherical, 43–70 × 37–70 (Fig. 3A–C; Fig. 4C, G, K). Prepharynx short or absent, 2–9 long, pharynx small, subspherical, 18–35 × 10–32, oesophagus 23–235 long. Intestinal bifurcation pre-equatorial, immediately anterior to cirrus sac. Caeca short, widely divergent, reaching anterior or middle level of ventral sucker (Fig. 3D). Testes 2 slightly symmetrical, postovarian, subspherical, right testis 26–69 × 21–67, left testis 29–49 × 22–57. Cirrus sac transverse, intercaecal, anterior of ventral sucker dorsally. Seminal vesicle elongate-oval. Prostatic cells large, numerous. Ejaculatory duct short. Genital pore oval, sinistrolateral to ventral sucker (Fig. 3A–C, Fig. 4G, K). Ovary dextral oval, adjacent to ventral sucker dorsally, 34–73 × 30–76. Oviduct short. Mehlis’s gland distinct, median, anteroposterior-ovarian. Laurer’s canal not observed. Eggs numerous, small, 9–20 × 5–12. Vitellarium in hindbody, comprised numerous small asymmetrical follicles. Excretory vesicle short, terminal, V-shaped; pore terminal (Fig. 3A–C).

### Metacercariae

[Measurements based on whole mounts of seven specimens and 3 specimens for SEM

(Fig. 3D; Fig. 4M–P).] Unencysted metacercaria, body oval (Fig. 3D), 210–284 × 73–131. Tegument with a few minute spines antero-posteriorly (Fig. 4M, P). Eyespot observed in the anterior region, located at the level of the pharynx and reaching the intestinal bifurcation (Fig. 3D). Sensory papillae scattered across the body tegument (Fig. 4M). Oral sucker terminal, subspherical, 25–54 × 40–77, with sensory papillae and recovered with spines (Fig. 4N). Ventral sucker post-equatorial, subspherical, 29–53 × 27–62, with sensory papillae covered with spines (Fig. 4O). Prepharynx short or absent. Pharynx small, subspherical, 4 × 3. Oesophagus 21 long. Intestinal bifurcation in forebody. Caeca short (Fig. 3A–C). Genital pore oval, sinistrolateral to ventral sucker. Primordia of testis elongate-oval, in tandem; ovary elongate-oval, anterior to the testes. Ootype 9 X 12 (n = 1). Excretory vesicle V-shaped.

### Taxonomic summary

*Microphallus basodactylophallus* (Bridgman, 1969) Deblock, 1971.

Type host: *Procyon lotor* Linnaeus.

Other hosts: *Oryzomys palustris* Harlan; *Eudocimus albus* Linnaeus; *Buteogallus* *urubitinga* Gmelin.

Type locality: Mississippi River, Louisiana, USA.

Other locality; Tlacotalpan, Veracruz, Mexico; Playa Paraíso, Tabasco; Tupilco, Tabasco, México.

### Phylogenetic analyses

**Fig. 5:**
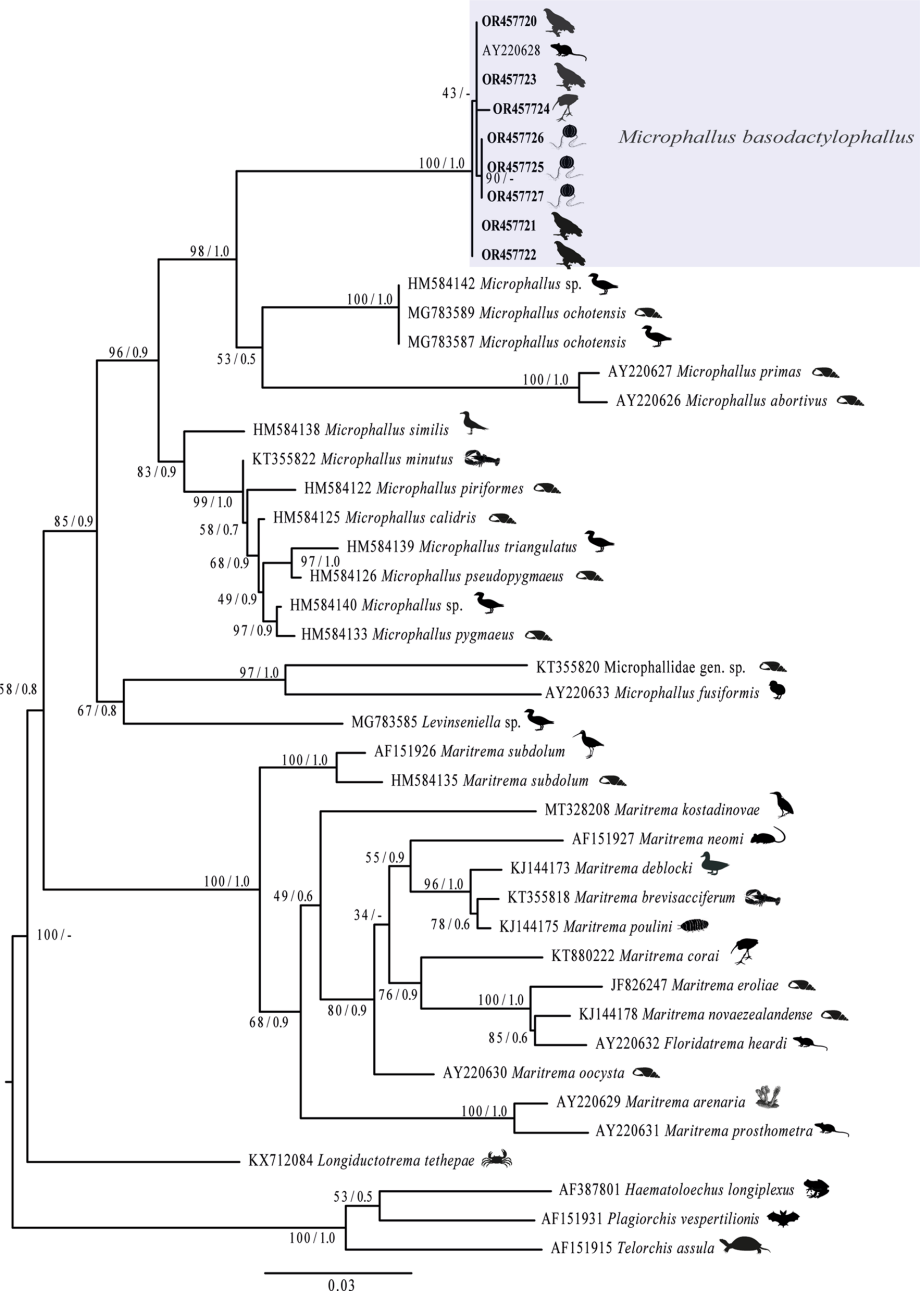
Consensus Bayesian inference and Maximum likelihood trees inferred with the LSU dataset. Numbers near internal nodes show ML bootstrap percentages/Bayesian posterior probabilities.

The newly generated sequences (five adults and three metacercariae) from the LSU were analysed together with 35 published sequences from 29 species, forming an alignment of 1,279 sites. The best evolutionary model was GTR+I+G. This dataset included species of the genera *Microphallus*, *Maritrema* Nicoll, 1907*, Levinseniella* Ward, 1901 and *Longiductotrema* Deblock & Heard, 1969 belonging to Microphallidae, plus sequences of three representatives belonging to *Haematoloechus* Looss, 1899*, Plagiorchis* Lühe, 1899 and *Telorchis* Lühe 1899 used as outgroups (Fig. 5), since they have a common ancestry with microphallids (see Kudlai et al., 2015; Galaktionov & Blasco-Costa, 2018). The newly obtained LSU sequences formed a fully supported, monophyletic assemblage, with that of *M. basodactylophallus* (GenBank: AY220628), recovered from the marsh rice rat (*Oryzomys palustris*) in the USA. This clade was sister to a lineage formed by several other congeners with high nodal support (Fig. 5). The interspecific genetic divergence estimated among the species of *Microphallus* spp., ranged from 1.1 to 7%, whereas the genetic divergence estimated among adults and metacercariae identified as *M. basodactylophallus* ranged from 0.0 to 0.06%.

## Discussion

To the best of our knowledge, two species of the genus *Microphallus* (*M. muellhaupti* Coil, 1956, and *M. trilobatus* Cable & Kuntz, 1951) have been recorded in Mexico (see Pérez-Ponce de León et al., 2007). The present finding of metacercaria and adults of *M. basodactylophallus* infecting ctenophores (*Pleurobrachia* sp.), white ibises (*E. albus*) and great black hawks (*B. urubitinga*) represent new hosts and locality records for this species of parasite expanding its geographic occurrence distribution from USA to southeastern Mexico. The species *M. basodactylophallus* was originally described in the racoon (*Procyon lotor*) from southern Louisiana (Bridgman, 1969). However, type specimens of

*M. basodactylophallus* were obtained through experimental infection of a rat (*Rattus norvegicus albinus* Berkenhout) (Bridgman, 1969) and, since its description, the parasite has been recorded in Florida, Mississippi, Alabama and Georgia, (all USA) (Heard & Overstreet, 1983), which may explain the intraspecific morphometric variation observed.

The life cycle of *M. basodactylophallus* is well known; i.e., the snails *Lydores parvula* (Guilding), *Littoridinops monroensis* Frauenfeld and *Hydrobia* sp., serve as the first intermediate hosts. The cercaria emerge, swim, and penetrate into crabs (blue crab *Callinectes sapidus* Rathbun, red‐jointed fiddler crab *Uca minax* (Le Conte), Atlantic marsh fiddler crab *Minuca pugnax* (Smith) and gulf marsh fiddler crab (*Minuca longisignalis*) Salmon & Atsaides), where metacercaria develop and encyst. Finally, the second intermediate host with metacercariae is eaten by the definitive host, in which the adult stage occurs (Bridgman, 1969; Heard & Overstreet, 1983). In the present study, unencysted metacercariae identified as *M. basodactylophallus* were found in the mesoglea of ctenophores of *Pleurobrachia* sp., with a prevalence value of 7% (376 examined hosts/26 hosts parasitized) and intensity of 1–6 per infected host. Metacercariae occur in a wide variety of planktonic and benthonic animals, such as ctenophores and hydromedusas, which serve as second intermediate hosts for digeneas of the superfamilies Gymnophalloidea, Hemiuroidea and Lepocreadioidea (Marcogliese, 1995; Martorelli, 2001; Cribb et al., 2003; Moradini et al., 2005). The evidence found in the present study suggests that the cercaria actively penetrate the ctenophore, losing their tails and persisting inside host’s mesoglea as unencysted metacercaria. The unencysted metacercaria of *M. basodactylophallus* in ctenophores could be consider as an accidental infection. However, is well known that ctenophores may be an important food in fish diet (Mianzan et al., 1996, 2001) and most likely, act as a trophic link for the parasites in the way to reach its definitive host.

The inclusion of molecular data in this study was crucial to link metacercariae and adults of *M. basodactylophallus* parasitizing invertebrates, mammals and aquatic birds from USA to southeastern Mexico. It was demonstrated that the body size, oral sucker, ventral sucker, pharynx, oesophagus, testes and eggs exhibit some morphometric variations (see Table 1). Therefore, the use of molecular markers is necessary to link development stages of digeneas, as well as for the description and delimitation of species of *Microphallus*. The present phylogenetic analyses performed with the LSU datasets confirmed that *Microphallus* is monophyletic and shares a common ancestor with *Maritrema* with low values of support. However, the near relationship between both genera is consistent with the results of previous phylogenetic analyses of microphallids (Kudlai et al., 2015; Galaktionov & Blasco-Costa, 2018).

The metacercariae and adults identified as *M. basodactylophallus* from ctenophores (*Pleurobrachia* sp.), white ibis (*E. albus*) and great black hawk (*B. urubitinga*) at the four locations in southeastern Mexico formed a well-supported monophyletic assemblage with other *M. basodactylophallus* from Florida, USA, with LSU sequences available in GenBank (AY220628) (Fig. 5). The intraspecific genetic divergence estimated among the isolates of *M. basodactylophallus* ranged from 0.0 to 0.6%. These values of intraspecific genetic divergence are higher than those previously reported for four isolates of *M. ochotensis* Galaktionov & Blasco-Costa, 2018, including two metacercariae (MG783588–MG783589) and two adults (MG783586–MG783587), and between a cercaria (KT355823) and a metacercaria (KT355823) of *M. minutus* Johnston, 1948, both of which showed zero genetic divergence of the LSU (Kudlai et al. 2015; Galaktionov & Blasco-Costa, 2018). Finally, the interspecific genetic divergence estimated among the species of *Microphallus* spp., ranged from 1.1 to 7%. The lowest genetic divergence was between *M. triangulatus* Galaktionov, 1984 (HM584139) and *M. pseudopygmaeus* Galaktionov, 1980 (HM584126) and the highest genetic divergence was between *M. primas* (Jägerskiöld, 1908) Stunkard, 1951 (AY220627) and *M. ochotensis* Galaktionov & Blasco-Costa, 2018 (MG783587 and MG783589). These values of interspecific genetic divergence are lower than those previously reported by Kudlai et al. (2016), which ranged from 6.5 to 11%.

The ecological evidence suggests that microphallids show low host specificity regarding the definitive host (see Caveny & Etges 1971; Deblock, 2008). In the present study, was found that *M. basodactylophallus* can parasitize both mammals and aquatic birds, favouring its distribution and dispersion along coasts in the Neotropical region. The unencysted metacercariae of *M. basodactylophallus* represent the first report of a microphallid in ctenophores from southeastern Mexico.

## Data Availability

Not applicable.
